# Patient variability in the blood-stage dynamics of *Plasmodium falciparum* captured by clustering historical data

**DOI:** 10.1186/s12936-022-04317-0

**Published:** 2022-10-26

**Authors:** Thiery Masserey, Melissa A. Penny, Tamsin E. Lee

**Affiliations:** 1 Swiss Tropical and Public Health Institute, Kreuzstrasse 2, 4123 Allschwil, Basel-Land Switzerland; 2 University of Basel, Basel, Switzerland

**Keywords:** Malaria, *Plasmodium falciparum*, Blood-stage infections, Patient variability, Cluster analysis, Mathematical model

## Abstract

**Background:**

Mathematical models provide an understanding of the dynamics of a *Plasmodium falciparum* blood-stage infection (within-host models), and can predict the impact of control strategies that affect the blood-stage of malaria. However, the dynamics of *P. falciparum* blood-stage infections are highly variable between individuals. Within-host models use different techniques to capture this inter-individual variation. This struggle may be unnecessary because patients can be clustered according to similar key within-host dynamics. This study aimed to identify clusters of patients with similar parasitaemia profiles so that future mathematical models can include an improved understanding of within-host variation.

**Methods:**

Patients’ parasitaemia data were analyzed to identify (i) clusters of patients (from 35 patients) that have a similar overall parasitaemia profile and (ii) clusters of patients (from 100 patients) that have a similar first wave of parasitaemia. For each cluster analysis, patients were clustered based on key features which previous models used to summarize parasitaemia dynamics. The clustering analyses were performed using a finite mixture model. The centroid values of the clusters were used to parameterize two established within-host models to generate parasitaemia profiles. These profiles (that used the novel centroid parameterization) were compared with profiles that used individual-specific parameterization (as in the original models), as well as profiles that ignored individual variation (using overall means for parameterization).

**Results:**

To capture the variation of within-host dynamics, when studying the overall parasitaemia profile, two clusters efficiently grouped patients based on their infection length and the height of the first parasitaemia peak. When studying the first wave of parasitaemia, five clusters efficiently grouped patients based on the height of the peak and the speed of the clearance following the peak of parasitaemia. The clusters were based on features that summarize the strength of patient innate and adaptive immune responses. Parameterizing previous within host-models based on cluster centroid values accurately predict individual patient parasitaemia profiles.

**Conclusion:**

This study confirms that patients have personalized immune responses, which explains the variation of parasitaemia dynamics. Clustering can guide the optimal inclusion of within-host variation in future studies, and inform the design and parameterization of population-based models.

**Supplementary Information:**

The online version contains supplementary material available at 10.1186/s12936-022-04317-0.

## Background

Malaria continues to be a global health priority. In 2020, the World Health Organization (WHO) estimated that malaria caused 241 million cases and 627,000 deaths worldwide [1]. *Plasmodium falciparum* was responsible for the majority of the morbidity and mortality associated with malaria [1]. Most cases and deaths occurred in the African region, and mainly affected children under 5 years old who had not yet developed an efficient adaptive immune response [1].

The *P. falciparum* life cycle involves several stages in two different hosts: within the insect vector (female *Anopheles* mosquito) and within the human host, including a liver-stage and blood-stage cycle [2, 3]. The dynamics of the blood-stage have a great influence on the epidemiology of malaria. For the individual, the morbidity and mortality of patients depend on the parasite density during the blood-stage [2–6]. For the population, the blood-stage dynamics influence the time that patients are infectious and their infectiousness to mosquitoes, which determine the transmission of malaria [2, 3, 7, 8].

The most detailed data of blood-stage dynamics of *P. falciparum* is from the malariatherapy studies [9]. The studies were conducted by the National Institutes of Health laboratories in the USA when malariatherapy was used to treat neurosyphilis (1931–1963) [9]. All patients were Afro-American adults suffering from neurosyphilis and had no previous exposure to malaria [9]. The dataset contains daily measurements of the *P. falciparum* density for 334 patients from the infection day until the day that they recovered, due to treatment or spontaneously [9]. This dataset highlighted the extreme inter-patient variability of parasitaemia density profiles during the blood-stage, for example, the natural infection lengths of patients varied between 37 and 405 days [9]. These variations are due to the complex interactions between the host and the parasite, variations among individual immune responses, and between parasite strains [10–20].

Many within-host mathematical models have investigated blood-stage dynamics of *P. falciparum*, and reproduced the parasitaemia dynamics of malariatherapy patients by considering the variations of host immune responses and parasite strains [21–28]. However, because of the extreme variation between individuals, predicting a parasitaemia profile for a specific patient generally relied on model parameters that were case-specific or stochastically chosen from assumed distributions. Furthermore, models were often calibrated to patient parasitaemia profiles using summary statistics which ignored individual variation across these statistics [21–28]. Consequently, some of these models cannot be used to predict patient a specific parasitaemia profile without knowing the parasitaemia profile of the patient beforehand. Moreover, a recent review and analysis highlighted that the outcomes of within-host models are likely not robust, as a slight variation of parameter value created totally different predictions [29]. This review suggested that the efficiency of within-host models to capture variation in infection dynamics depends more on the selection of the best-fitting simulations than the value of the parameters [29].

Including patient variation of within-host dynamics in transmission models for malaria, at the population level, is also challenging. Compartmental models, such as [30, 31], assume that all hosts have homogenous within-host dynamics. Therefore, their use is limited when estimating the effect of control strategies that directly impact the asexual stage. In contrast, individual-based models (IBMs) include individual variability of blood-stage dynamics [32]. This can make them computationally difficult to simulate [32], and they require many parameters, so fitting to data is complex [33, 34].

This paper aimed to find the balance between assuming a homogeneous population and modelling each individual specifically. Attributes that capture the essential information of the parasitaemia profile for an individual were selected. Clustering based on these attributes provided centroid values, so that most of the individual variation could be accounted with few additional parameters.

First, clusters of patients with similar full parasitaemia dynamics were identified. From the malariatherapy studies described above [9], only 35 patients had a complete parasitaemia profile. Thus a second cluster analysis was performed to identify patients with a similar first wave of parasitaemia. For this analysis, the sample size increased to 100 individuals and thus accounted for more individual variation. Then, the ability of the clusters to capture the variability of within-host dynamics was evaluated using within-host models [21, 22]. Two within-host models were used: one that predicted the parasitaemia density for a patient throughout an untreated infection (Molineaux et al. [21]), and one that predicted the first wave in the parasitaemia density (Dietz et al. [22]). Parasitaemia profiles were predicted using case-specific parameters (as in the original papers), using cluster-specific parameters, and lastly, using parameters derived from the means of the whole dataset. These predictions were compared to assess the ability of the clusters to capture the variability of within-host dynamics.

Moreover, the distribution of non-parasitaemia attributes, such as the sex of the patient and the strains used for the infection, were compared across the clusters. Identifying clusters of patients with similar parasitaemia dynamics will improve the inclusion of patient within-host variation in future within-host and population-based models whilst remaining computationally inexpensive.

## Methods

In this section, the methods for both datasets and clustering analysis are provided separately. The first section describes the data, the potential attributes which could be used to cluster the data, and the clustering method. Then, the second section describes the method to evaluate the clusters ability to capture patients variability using within-host models [21, 22]. The last section describes the method used to investigate how non-parasitaemia attributes, such as the sex of the patient and the strains used for the infection, correlate with the clusters.

### Clustering the full parasitaemia profile

#### Data

Data from patients that had a complete natural parasitaemia dynamic profile, not affected by curative treatment, were used to identify clusters of patients with a similar natural parasitaemia dynamic [21]. In the malariatherapy dataset, 35 patients fulfilled these criteria. In this subset, 16 patients received a low-dose treatment (quinine, chloroguanide, or chloroquine). However, these patients were included in the analysis because the treatment had only a limited and short effect on the parasitaemia dynamic [21]. Patients were infected either by inoculation of infected blood (18 cases) or by mosquito bites (17 cases). Patients were inoculated with different strains of *P. falciparum*: 17 with El Limon, 17 with Sante Cooper, and 1 with the McLendon strain [21]. Parasitaemia levels were measured daily by microscopy [the detection limit was equal to ten parasitized red blood cells per microlitre (PRBC/µl)].

#### Clustering analysis

To identify clusters of patients with similar natural parasitaemia dynamics, the 35 patients were clustered based on nine key features used by several modelling papers to summarize a patient’s parasitaemia profile (Table 1) [21]. Using these features as attributes, the data was clustered with all possible combinations of pairs of attributes. Clustering with three or more attributes was not feasible because the dataset was relatively small, so three or more attributes led to unreliable clusters.

**Table 1:** Key features of the full parasitaemia profile

	Key feature	Definition
(i)	The initial slope	The slope of a linear regression line through the log densities from the first positive slide to the first local maximum (per day)
(ii)	Number of local maximums	A measurement was a maximum if its density was higher than the densities at times t: (t−6), (t−4), (t−2), (t + 6), (t + 4), (t + 2) (no units)
(iii)	Density at the first maximum	Density at the first maximum (log Parasitized Red Blood Cells (PRBC) per microlitre)
(iv)	The slope of local maximums	The slope of a linear regression line through the log densities of the local maxima (per day)
(v)	The geometric mean	The geometric mean of the intervals between consecutive local maximums (days)
(vi)	The standard deviation	The standard deviation of the logs of the intervals between consecutive local maximums (no units)
(vii)	The proportion of positive in the first interval	The proportion of measurements higher than zero in the first half of the interval between the first and last positive day (no units)
(viii)	The proportion of positive in the second interval	The proportion of measurements higher than zero in the second half of the interval between the first and last positive day (no units)
(ix)	The last positive day	The last days that the patient had a parasitaemia higher than 0 (days)

A description of the features used by [21] to summarize patient’s parasitaemia profiles

Clustering assumed that the features were independent, which, strictly speaking, they were not in this case, as all nine features arose from the parasitaemia profile. Nonetheless, clustering remained a relevant analysis technique since the pairwise dependencies between the features were not straightforward. For all combinations of two standardized key features, clusters of individuals were identified using a finite mixture model fitted by expectation maximization [35]. This method was used because it allowed for more variance in the size and shape of the clusters and more variance in the range of the key features compared to classical clustering approaches, such as k-means clustering.

The optimal number of clusters was determined for each combination using the Bayesian Information Criterion index (BIC) [35]. The optimal number of clusters is a balance between a good fit (how well the cluster represented each patient which belonged to that cluster) and the number of clustering parameters (number of clusters). Later, when using the cluster centroids to predict the parasitaemia profiles, the effect of having more than the ‘optimal’ number of clusters was also explored.

Finally, the robustness of the clusters was assessed using leave-one-out analysis. The leave-one-out analysis involved performing the clustering without one patient and then assigning this patient to a cluster based on the chosen attributes. This process was repeated for all patients. A successful cluster assigned the left-out patient to the cluster to which they were assigned when they were included. The proportion of patients correctly assigned to their cluster was referred as the robustness score, such that a score of unity was perfect.

#### Evaluating the clusters

Molineaux et al*.* [21] considered many biological aspects of the dynamics and their variability: intraconal antigenic variation (50 variants); variation of the variant baseline growth rate (variable among variant and host); innate immune response (variable among host); acquired variant-specific immune response corresponding to the variant-specific antibodies that target *Plasmodium falciparum* erythrocyte membrane protein 1 (PfEMP1) expressed on the red blood cell membrane; acquired variant transcending immune response (variable among host) representing the antibodies that target merozoite surface proteins; and the measurement error [21]. Molineaux et al. [21] predicted an individual parasitaemia density on a given day based on the parasitaemia density 48 h previously, the multiplication rate of the antigenic variants, and the immune responses. The strength of the immune responses was determined by two critical parasite densities that were specific to each patient:

- P_c_ was the host critical density that determined the innate immune system strength (named P_c_* in Molineaux et al. [21]). It was based on the first maximum of parasitaemia ((iii) from Table 1),
- *P*_*m*_ was the host critical density that determined the transcending adaptive immune system strength (named P_m_* in Molineaux et al. [21]). It was based on the infection length of the patients ((ix) from Table 1).

For each patient, the multiplicatio﻿n rates for the variant’s baseline growth rates were sampled from a normal distribution centred around 16 [21]. The final simulated profile was determined by comparing (using chi-squared) the nine key features (Table 1) from 50 simulated profiles with the key features from the actual patient profile. Note that Molineaux et al*.* [21] used case-specific data when first defining *P*_*c*_ and *P*_*m*_ and again when choosing from the 50 simulated profiles.

The novelty of this approach is to predict the parasitaemia dynamic of patients based on their respective clusters by parameterizing the model of Molineaux et al. [21] using cluster-specific parameter values for *P*_*c*_ and *P*_*m*_ instead of patient-specific parameter values. This means that all patients from the same cluster had the same parasitaemia profile based on the centroid values of that cluster. Note that the strength of the acquired variant-specific immune response in the model of Molineaux et al. [21] was not patient-specific and was, therefore, not relevant for clustering. For comparison, the model was also parameterized to each patient, replicating Molineaux et al. [21] (which corresponded to parameterizing the model to 35 clusters that each contain only one patient), and to all patients (which corresponded to parameterizing the model to one cluster that contains all patients). For each parameterization, the same methodology as Molineaux et al. [21] was followed except for the parameterization step and the selection of the best simulation (Table 2). Then, for each parameterization, the absolute error in the model’s prediction of the infection length, the first parasitaemia maximum of each patient, and a weighted error of these two errors was calculated. The weighted error was defined as the sum of the standardized error of the infection length and the standardized error of the log of the first parasitaemia maximum.

**Table 2:** The different parameterizations for the model of Molineaux et al. [21]

Model parameterized to:	Parameterization	The key features used to select the best simulation
All patients	The model was parameterized to all patients without using case- or cluster-specific parameters. The values of the two critical densities were the mean values of the 35 patients’ case-specific parameters	Compare the simulations with the mean values from the 35 patients
Each cluster	The model was parameterized to each cluster identified in the cluster analysis, using two cluster-specific parameters, calculated based on centroids values of the identified clusters	Compare the simulations with the centroids values of the clusters
Each patient	The model was parameterized to each patient using the two case-specific parameters, as defined in Molineaux et al. [21]	Compare the simulations with the observed values of each patient

Methods used to parameterize the two critical parasitaemia densities, P_c_ and P_m_ used in Molineaux et al. [21] when the model was parameterized to each patient, or each cluster, or all patients

### Clustering analysis of first parasitaemia wave

#### Data

The same subset of patients used by Dietz et al. [22] was selected from the full malariatherapy dataset to identify clusters of patients that have a similar first wave of parasitaemia*.* This subset fulfilled the following criteria: The maximum of the first wave was the absolute maximum of the case, the patient had no curative or suppressive treatment, the patient had no missing parasitology data on even days, the days in which the patient had a fever were known, the patient had at least five parasitaemia measurements, the first observed density was less than 320 PRBC/µl, the log(maximum /last density) was higher than 0.6, and the log(last/penultimate density) was lower than 0.3 [22]. These criteria were fulfilled by 100 patients. Patients were either infected by infected blood inoculation (67 cases) or by mosquito bites (33 cases) [22]. Patients were infected with different strains of *P. falciparum*: 50 patients with McLendon, 25 with El Limone, 21 with Sante Cooper, 3 with Colombia, and 1 with the Costa strain [22]. Parasitaemia levels were measured daily by microscopy (detection limit equal to 10 PRBC/µl) [22].

#### Clustering analysis

To identify clusters of patients with a similar first wave of parasitaemia, the 100 patients were clustered using five key features that summarized the dynamics of the first wave of parasitaemia, and were used as case-specific parameters by Dietz et al*.* [22] (Table 3). As in the previous section, for all pairwise combinations of standardized key features, clusters of individuals were identified using a finite mixture model fitted by expectation maximization [35]. As before, the optimal number of clusters was determined for each combination using the BIC, and the robustness of the clusters was assessed using leave-one-out analysis [35].

**Table 3:** Key features of the first parasitaemia wave

	Key feature	Definition
(i)	Multiplication factor	The 2-day multiplication factor by which the parasite density was multiplied in the absence of any immunity (no units)
(ii)	Critical effective density	The critical parasite density at which the current multiplication factor was reduced by 50% (PRBC/µl)
(iii)	Cumulative effective density	The cumulative parasite density at which the current multiplication factor was reduced by 50% (PRBC/µl)
(iv)	Delay of the onset of adaptive immunity	The delay required by the adaptive immunity to become effective (days)
(v)	Density at the first maximum	Density at the first maximum (PRBC/µl)

A description of the features used by [22] to summarize patients first parasitaemia waves

#### Evaluating the clusters

The model from Dietz et al*.* [22] was used to assess how well each cluster predicted the first wave of parasitaemia for each individual. This model reproduced the first wave for the same 100 patients. Dietz et al. [22] simplified the model from Molineaux et al*.* [21] to consider only one PfEMP1 variant, and thus, instead of modelling three immunity components, only two were required: innate immunity and acquired immunity. Dietz et al*.* used four case-specific parameters to reproduce the natural first wave of patients (Table 4) [22].

**Table 4:** Case-specific parameters used by Dietz et al. [22]

Notation	Definition		Calculation
m	Multiplication factor of the parasite		Maximum two-day multiplication factor by which the parasite density was multiplied by during the first wave (no units)
P_c_	Critical effective density that determines the strength of the innate immune response		Critical parasite density at which the current multiplication factor was reduced by 50% (PRBC/µl)
P_m_	Cumulative effective density that determines the strength of the adaptive immune response		Cumulative parasite density at which the current multiplication factor was reduced by 50% (PRBC/µl)
δ0	Delay of the onset of adaptive immunity		Estimated time of the first inflection points of the parasitaemia density (days)

Notation, definitions and calculations of the case-specific parameters used by Dietz et al. [22]

To predict the typical first parasitaemia wave of patients based on their respective cluster, the model was parameterized to the clusters using the cluster-specific versions of these parameters (Tables 4 and 5). Dietz et al*.* [22] used the Powell Hill-climbing algorithm to calculate the case-specific parameters. Here, the parameters were directly calculated as defined in Table 4. To improve the accuracy of the prediction, the calculation of the cumulative effective density (*P*_*m*_) was modified: 4830 divided by the ratio between the maximum density and the last density of the first parasitaemia wave. The improvement in the accuracy of the model is detailed in Additional file 1.

**Table 5:** The tested parameterizations for the model of Dietz et al. [22]

Model parameterized to	Parameterization
All patients	The model was parameterized to all patients without using any case or cluster-specific parameters. The values of the four constants were the means of the case-specific parameters over all patients
Each cluster	The model was parameterized to each cluster identified in the cluster analysis, using four cluster-specific parameters based on centroids values of the clusters
Each patient	The model was parameterized to each patient by using the four case-specific parameters defined by Dietz et al. [22]

Methods used to parameterize the model from Dietz et al. [22] to all patients, each cluster, or each patient

As before, the model was parameterized to each patient (which corresponded to parameterizing the model to 100 clusters that each contain only one patient) and to all patients (which corresponded to parameterizing the model to one cluster that contains all patients) (Table 5). The error of the predicted parasitaemia measurements was calculated for each patient.

### External attributes

For both clustering analyses, the distributions of all available external attributes across the identified clusters were examined: the sex of patients, the use of a repressive treatment, the strains used for the infection (Santee Cooper, Mc Lendon, El Limon, Colombia, Costa), and the method of infection (blood induction or sporozoite induction). For the full parasitaemia profile, the patient’s fever and gametocyte profiles were additionally compared for each cluster. This comparison was not made for the first wave of parasitaemia, as many patients received treatment that biased their fever and gametocyte profile.

## Results

### Clustering analysis of parasitaemia profile

**Fig. 1:**
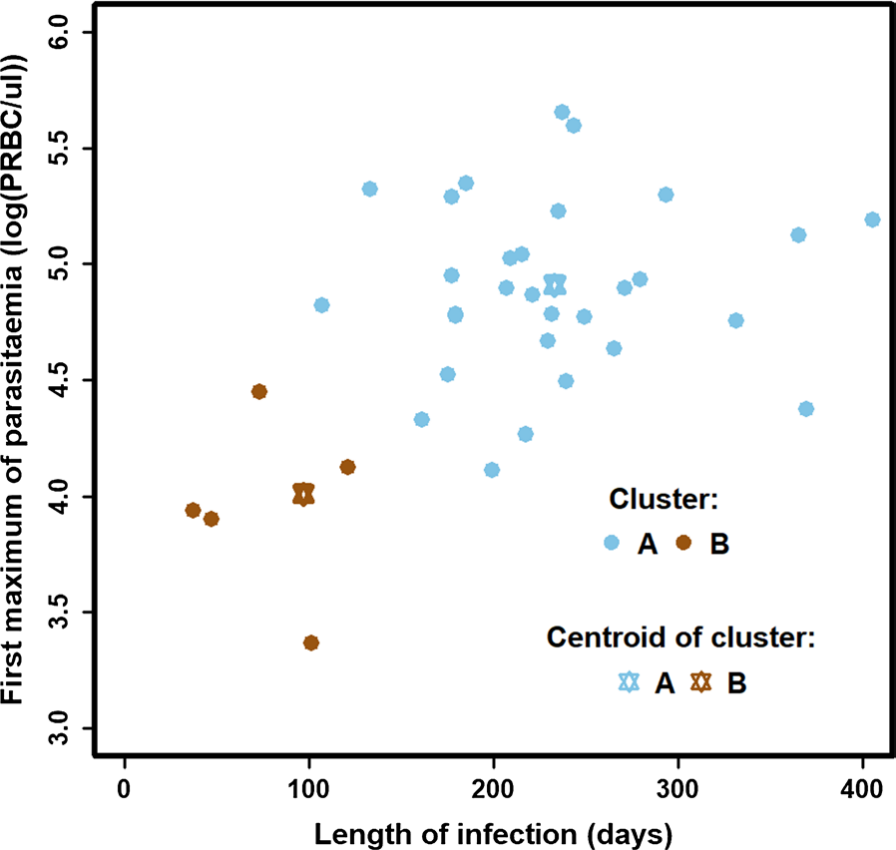
Clusters of the full parasitaemia profile. Clusters of the full parasitaemia profile of 35 patients based on the strongest clustering identified by trying all pairwise combinations of attributes listed in Table 1

The strongest pairwise clustering was found to contain the length of infection (ix) and the density of the first maximum of parasitaemia (iii) (Fig. 1). The optimal number of clusters defined by the BIC was two (robustness score = 32/35 = 0.91). Patients were grouped in a big cluster of 30 patients (cluster A) and a small cluster of five patients (cluster B) (Fig. 1). Patients in cluster A had a long infection length and high density at the first parasitaemia maximum (centroids value of cluster A: 233 days, 4.91 log(PRBC/µl). In contrast, patients in cluster B had a shorter infection length and a lower first maximum of parasitaemia (centroids value of cluster B: 97 days, 4.01 log(PRBC/µl). This indicates that patients with a higher first peak of parasitaemia tended to be infected for a longer period.

The clustering results supported the modelling choice in Molineaux et al. [21] to use parameters P_c_ and P_m_, which are based on the first parasitaemia maximum and the last positive days, respectively [21]. Parameterizing the model from Molineaux et al. [21] to the clusters required simply using the centroid values of the clusters instead of case-specific values for P_c_ and P_m_

#### Prediction of patient’s parasitaemia profiles

**Fig. 2:**
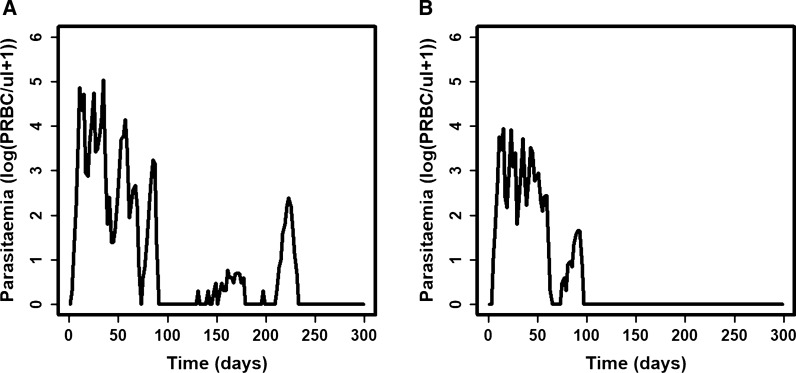
Predicted parasitaemia profile. Prediction of the typical parasitaemia profile for patients from **A** cluster A and **B** cluster B using the model of Molineaux et al. [21] parameterized to the centroid values of the clusters

The typical parasitaemia profiles for patients within each cluster were for each cluster predicted (Fig. 2). To analyze the effect of the number of clusters on the error, the typical parasitaemia profiles from clustering the data to 3, 4, 6 and 8 clusters were also predicted.

**Fig. 3:**
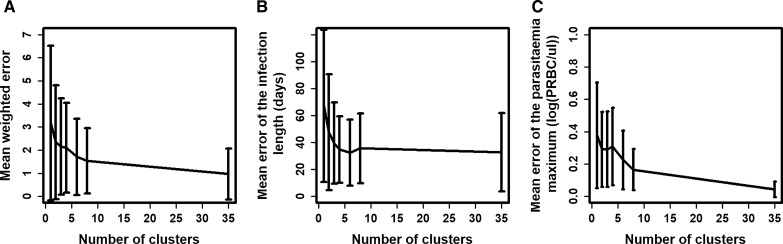
The number of clusters and the prediction errors for the patient’s full parasitaemia profile. The relationship between the number of clusters (to which the model was parameterized) and the **A** mean weighted error, which is the sum of the standardized error of the infection length and standardized error of the log of the first parasitaemia maximum **B** mean error of the infection length, and **C** the mean error of the parasitaemia maximum of the 35 patients. The vertical lines represent the standard deviation of these errors

Generally, the combined mean error decreased when the number of clusters increased, as did its standard deviation (Fig. 3A). With only one cluster, the mean weighted error was 3.17. By parameterizing the model to the two identified clusters, the mean weighted error was reduced to 2.34 (reduction of 26.2% compared to one cluster). This decrease was the biggest drop in the weighted error, which arose from adding only one additional cluster. For example, a third cluster only slightly reduced the mean weighted error further to 2.15 (reduction of 32.2% compared to one cluster). Nevertheless, as expected, the model parameterized to the 35 clusters had the minimum error, with a mean weighted error of 0.97 (reduction of 69.4% compared to one cluster).

The errors for the two parasitaemia values, the error in the infection length and the first maximum of parasitaemia, were reduced by parameterizing the model to the two identified clusters (Fig. 3B and C), compared to using one cluster only. The error for the infection length predictions was reduced slightly more (reduction of 29.1% compared to one cluster) than the error for predictions of the maximum of parasitaemia (reduction of 23.6% compared to one cluster). Note that the variation in the infection length over all patients was higher than the variation in the first peak of parasitaemia, suggesting that the variation of the adaptive immune response is stronger than the variation of the innate immune response. In contrast, when comparing the model parameterized to one cluster over the model parameterized to 35 clusters, the error for the first parasitaemia maximum was reduced more (error reduction of 88.6% compared to one cluster) than the error for the infection length of patients (error reduction of 51.0% compared to one cluster). This highlights that Molineaux et al. [21] captured the first parasitaemia maximum better than the infection length of patients.

**Fig. 4:**
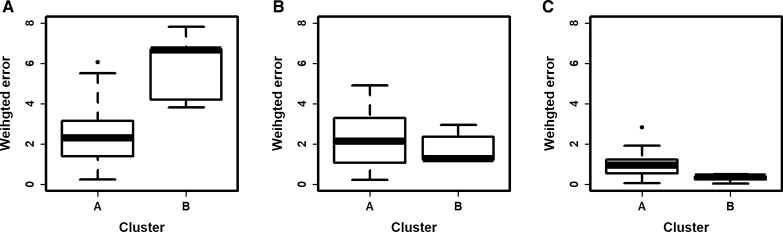
Distribution of the weighted error among clusters for each parameterization. Distribution of the weighted error of patients in clusters A or B when the model was parameterized to **A** 1 cluster, **B** 2 clusters, and **C** 35 clusters

The weighted error was examined separately for clusters A and B under the three different parameterizations. When the model was parameterized to 1 cluster, the weighted error was higher in cluster B (mean error 5.83) than in cluster A (mean error 2.48), meaning that patients in cluster B (the smaller cluster) were considerably different to the overall average (Fig. 4A). This hypothesis was also supported by the drop in the weighted error (from 5.83 to 1.81) for cluster B once the parameterization considered these patients separately (Fig. 4B). The weighted error of cluster A dropped when the number of clusters increased from one to two, but this drop was less dramatic (from 2.48 to 2.26). As expected, parameterizing to 35 clusters reduced the error further (mean error for cluster A: 0.94, cluster B: 0.32) (Fig. 4C). In this case, the reason that cluster A had a higher weighted error may be due to more inter-patient variation [36].

#### Comparing non-parasitaemia attributes and clusters

When comparing non-parasitaemia attributes amongst the two clusters, patients from cluster B (the cluster with low parasitaemia density) did not receive treatment (Additional file 1: Figure S3), which was expected because treatment was only given to patients with a high parasitaemia load. Moreover, there was no relationship between clusters and the parasite strain, nor the method of infection, meaning that these factors did not have a strong influence on the within-host dynamic (Additional file 1: Figure S3). However, the ten female patients (out of 35) all belonged to cluster A, meaning that from this dataset, all female patients experienced a high parasitaemia load and long infection length (Additional file 1: Figure S3).

In addition, the gametocyte profile of patients also varied by clusters. Patients from cluster A had a higher maximum peak of gametocyte density and number of days with detected gametocytes (Additional file 1: Figure S4). This observation was probably because parasitaemia density and gametocyte density are related, and the fact that patients from cluster A had a longer infection length and higher parasitaemia than patients from cluster B. In addition, these results could also be because some patients in cluster A received a low-dose treatment that could have triggered the generation of gametocytes. This result highlighted that patients from cluster A were probably more infectious than patients from cluster B.

There was also a straightforward relationship between the clusters and the fever of the patients. Patients from cluster A generally experienced fever for a longer period, and they had a higher maximum fever temperature than patients in cluster B (Additional file 1: Figure S5). This was because patients from cluster A had a longer infection length and a higher parasitaemia first peak (the fever often peaks at the beginning of the infection). These results indicate that patients from cluster A were more likely to suffer from severe symptoms than patients from cluster B.

### Clustering analysis of the first parasitaemia wave

The pair of attributes that gave the strongest clustering was the cumulative effective density (iii) and the maximum density of parasitaemia (v) (see Table 3 for definitions). In Dietz et al*.* the cumulative effective density (iii) captured the strength of the adaptive immune response, and thus determined the decreasing slope of the first parasitaemia wave [22]. The parasitaemia maximum (v) depended on both the multiplication factor of the parasite, and the host innate immune response [22]. Therefore, the clustering analysis confirmed that all the different shapes of the first parasitaemia waves were captured using these key features.

**Fig. 5:**
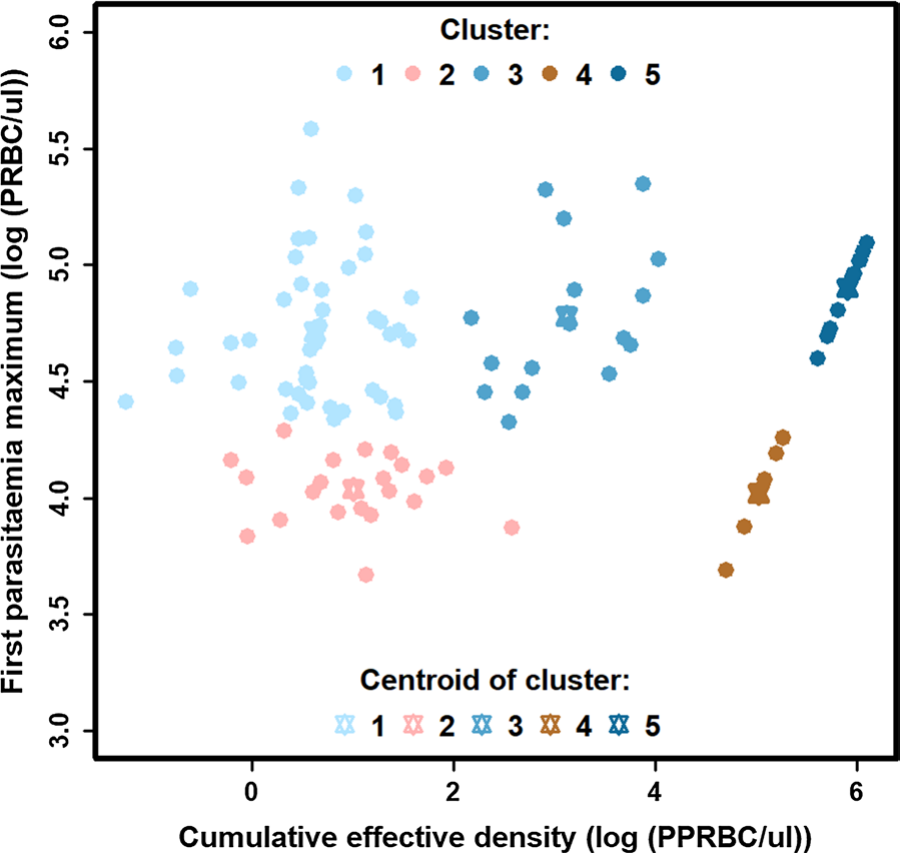
Clusters of the first parasitaemia wave. Clusters of the first parasitaemia wave of 100 patients based on the strongest clustering identified by trying all pairwise combinations of attributes listed in Table 3, where a preliminary division by the first peak of parasitaemia (higher than 4.3 log(PRBC/µl)) or a small first peak of parasitaemia (lower than 4.3 log(PRBC/µl) was performed

Due to the high variability of the data, the data were preliminary divided based on the result of the first cluster analysis. Recall that the first cluster analysis highlighted that patients either have a large first peak of parasitaemia (higher than 4.3(log(PRBC/µl)) or a small first peak (lower than 4.3 log(PRBC/µl)). After this preliminary division, two independent cluster analyses were performed on the two subsets. Two clusters, clusters 2 and 4, were identified in the subset of patients that had a low first peak of parasitaemia (centroids cluster 2: 4.04 log(PRBC/µl), cluster 4: 4.02 log(PRBC/µl) (Fig. 5). Three clusters, clusters 1, 3 and 5, were identified in the subset of patients that had a high first peak of parasitaemia (centroids cluster 1: 4.71 log(PRBC/µl), cluster 3: 4.78 log(PRBC/µl), cluster 5: 4.90 log(PRBC/µl)) (Fig. 5). Clusters 1, 2, 3, 4, and 5 grouped 45, 21, 16, 6, and 12 individuals, respectively. Moreover, in both subsets, some clusters had a low centroid value of the cumulative effective density (cluster 1: 0.63 log(PRBC/µl), cluster 2: 1.00 log(PRBC/µl)), and some clusters had a high centroid value of cumulative effective density (cluster 4: 5.02 log(PRBC/µl), cluster 5: 5.90 log(PRBC/µl)). In the subset of high first peaks of parasitaemia (clusters 1, 3 and 5), one cluster had a medium centroid value for the cumulative effective density (cluster 3: 3.12 log(PRBC/µl)).

#### Predictions of patients’ first parasitaemia wave

**Fig. 6:**
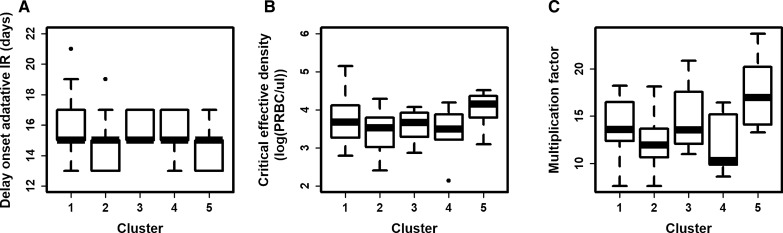
Distribution of key characteristics among clusters. Distribution of **A** the delay of the adaptive immune response (days), **B** critical effective density (log(PRBC/ul)), and **C** parasite multiplication factor (no units) across the identified clusters

The model from Dietz et al*.* [22] was parameterized to the five identified clusters. For each cluster, the cumulative effective density was equal to the cluster centroid value. The three other parameters (Table 4) were estimated as the median parameter values of patients belonging to the cluster. Note that the onset of the adaptive immunity did not vary across the clusters (Fig. 6A). However, as expected, clusters of patients with a lower maximum density of parasitaemia had a lower multiplication rate and critical effective density (which captured the strength of the innate immunity) (Fig. 6B and C).

**Fig. 7:**
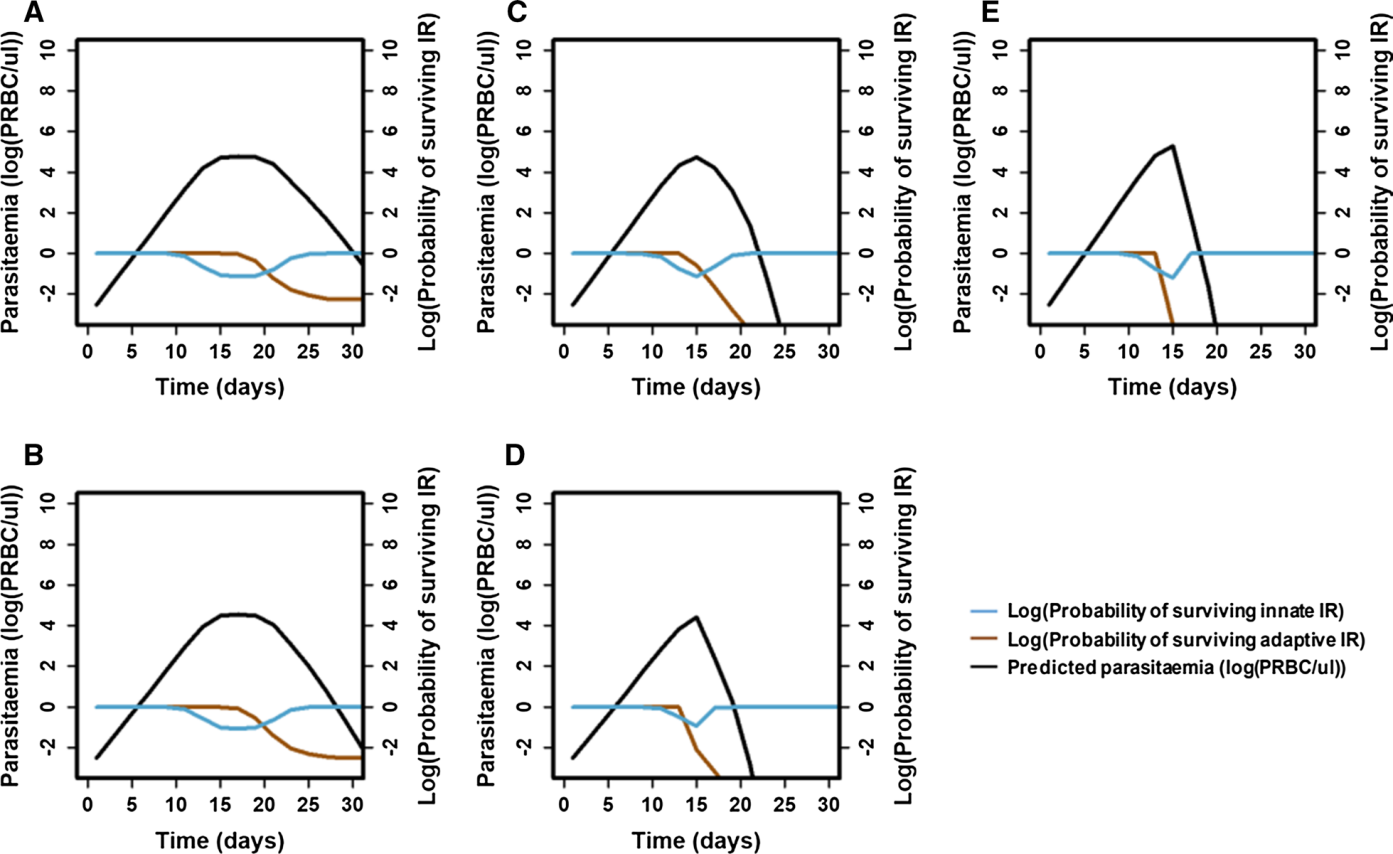
Predicted dynamic of the first wave of parasitaemia. The predicted first wave of parasitaemia and immune response (IR) dynamics for patients of each cluster: **A** 1, **B** 2, **C** 3, **D** 4, and **E** 5. The subplots are arranged corresponding to the clustering results in Fig. 5

As expected, the predicted maximums of the first parasitaemia wave of patients from clusters 2 and 4 were smaller (4.51, 4.48 log(PRBC/µl) respectively) than the maximum parasitaemia of patients from clusters 1, 3, and 5 (4.77, 4.74, 4.8 log(PRBC/µl) respectively) (Fig. 7). In addition, the typical first parasitaemia wave for patients in clusters 1 and 2 were long (39 and 35 days respectively) (Fig. 7A and B). Their first parasitaemia wave was characterized by a strong innate immune response, active for a long period, and a progressive increase of the adaptive immune response, which caused a progressive decrease in the parasitaemia density. In contrast, the typical first parasitaemia wave for patients in clusters 4 and 5 were short (23 and 21 days, respectively) (Fig. 7D and E). Their adaptive immune response became highly efficient quickly, which caused a rapid reduction of the parasitaemia density. Consequently, the innate immune response was not activated for a long time. Finally, patients in cluster 3 were between these two behaviours with a typical first wave lasting 27 days (Fig. 7C).

#### Prediction errors

**Fig. 8:**
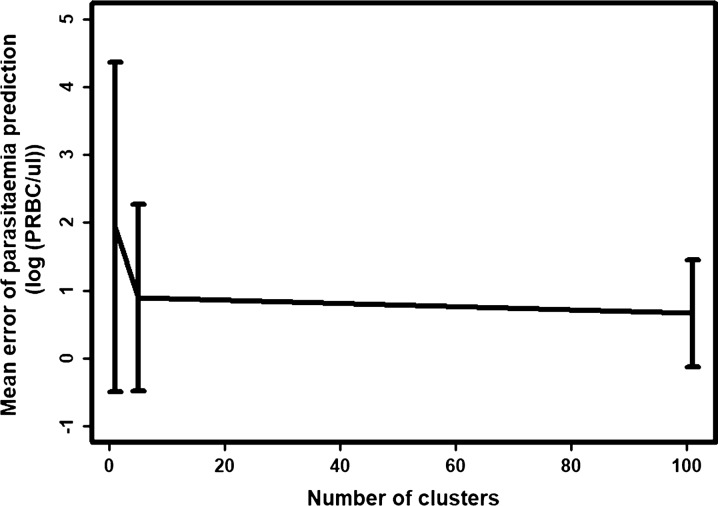
Relationship between the number of clusters and the prediction errors of the patient’s parasitaemia first wave. The relationship between the number of clusters to which the model was parameterized, and the mean error of the prediction of patient’s parasitaemia measurement (log(PRBC/ul)). The vertical lines represent the standard deviation of these errors

The error in the predictions of the model from Dietz et al. [22] for each patient was examined. The model was parameterized to each patient (100 clusters), to the cluster centroids (5 clusters), and to all patients (1 cluster). As expected, the error of patient’s parasitaemia measurement decreased as the number of clusters increased (Fig. 8). When the model was parameterized to all patients, the mean error was 1.93 (log(PRBC/µl)). When the model was parameterized to the five clusters, the mean error was greatly reduced (mean error 0.90 (log(PRBC/µl)), and had a similar value to when the model was parameterized to each patient (mean error 0.66 (log(PRBC/µl))). These results mean that the five clusters could effectively capture the variation of the first wave.

**Fig. 9:**
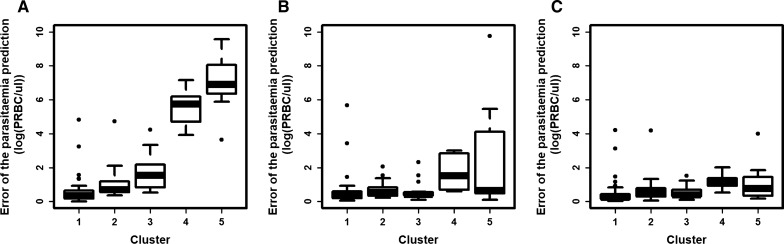
Distribution of the error in the predicted parasitaemia first wave among clusters for each parameterization. Distribution of the error of the predicted parasitaemia measurements (log(PRBC/ul)) across the five identified clusters when the model was parameterized to **A** 1, **B** 5 clusters, and **C** 100 clusters

When the model was parameterized using the overall means (one cluster), the error was higher for clusters 4 and 5 than for other clusters (Fig. 9A). When the model was parameterized to the five clusters, the error was reduced in every cluster (Fig. 9B). However, the distribution of the error was higher for clusters 4 and 5 than for the other clusters. This was also the case when the model was parameterized to each patient (Fig. 9C). This suggests that although clusters 1 and 2 were considerably larger than cluster 4, cluster 5 had more variation.

#### Comparing non-parasitaemia attributes and clusters

When comparing non-parasitaemia attributes amongst the five clusters, the results agreed with the analysis on the smaller dataset (Additional file 1: Figure S6). That is, the strains used to infect patients and the method of infection did not vary between the clusters of the first wave of parasitaemia. In addition, as with cluster B from the previous analysis, all female patients belonged to clusters characterized by a high maximum of parasitaemia (clusters 1, 3, and 5).

## Discussion

This study performed cluster analyses on two datasets of patients’ parasitaemia profiles based on key features which summarized the strengths of the innate and adaptive immune responses. The first dataset was the full, untreated, parasitaemia profile, and the second was the first wave of parasitaemia. The analysis provides insights in itself. For example, the clustering analysis of the full parasitaemia profile identified a smaller group of patients whose peak parasitaemia density and infection length were not well represented by the overall means. With regards to quantifying recovery and transmission rates in a population, the analysis provided reasonable estimates that largely account for heterogeneity without overloading population models with a set of parameters for each individual. Below the implications of the study are detailed.

Two clusters were identified when analysing the parasitaemia dynamics over a full infection: a large cluster of 85% of patients that had a long infection length and a high parasitaemia maximum (cluster A), and a small cluster of 15% of patients that had a short infection length and a low maximum of parasitaemia (cluster B). These results suggest that patients with a small first wave tend to have shorter infections. According to previous studies and models, the height of the first peak of parasitaemia, and the length of infection, are attributes that capture the strength of the patients innate and adaptative immunity [17, 21, 29, 37]. Consequently, these results highlighted that patients have variable immune response strengths, as reported in previous studies [10–12, 17–20], and suggest that patients with a strong innate immune response tend to have a strong adaptive immune response too.

Only two clusters were sufficient to capture the extreme variability of the full, untreated, parasitaemia profiles, and thus patients’ parasitaemia profiles were predicted without using case-specific data. Comparing the parameterization of one cluster to two clusters showed that only one extra cluster reduced the weighted error by 26.29%. The weighted error was especially reduced for patients with a short infection length and a low maximum of parasitaemia (cluster B). Therefore, patients in this cluster were not well represented by overall mean values. Exploring the effect of dividing the dataset into more clusters shows that it would be necessary to include many more clusters (6–8 clusters in total) to reduce the error further. However, additional parameters would increase the complexity of a model. Therefore, it is most efficient to consider only two clusters to capture within-host variation in a simple mechanistic within-host model, and to inform different recovery rates in a population-based model.

When the model from Molineaux et al*.* [21] was parameterized to each patient, the error of the infection length was high compared to the error of the first peak of parasitaemia. Therefore, the model from Molineaux et al. [21] is better at capturing the variation in the first peak of parasitaemia than the variation in infection length. This accuracy difference is probably because of the model stochasticity for the multiplication rate. Consider that for each patient, 50 simulations were run with different values for the multiplication factor. The resulting profiles had similar values for the parasitaemia maximum. However, the range for the predicted infection length was highly variable. Therefore, predicting the patient infection length was more sensitive to the parasite multiplication rate. This suggests that the accuracy of the predicted infection length in Molineaux et al*.* [21] is driven more by selecting the best simulation, as opposed to parameterizing the model to the data. The same conclusion was reached by a recent review of current within-host models [29] and a recent modelling study [25], which showed that a slight variation in a parameter caused a significant variability of infection length. Note that when the Molineaux et al*.* [21] model was used, a similar prediction error of the first parasitaemia maximum (0.037 log(PRBC/µl)) was obtained, but the predicted infection length of patients was on average 25 days closer to the actual infection length. This difference may have arisen from the use of different parasite multiplication factors, or they may have selected from the 50 simulations with more weighting on matching the infection length.

The resolution of the analysis was increased by clustering patients only on the first wave of parasitaemia. As in the first analysis, a minority of patients had a low first wave, and a majority of patients had a high first wave. Patients with a small first wave were clustered in a manner that separated those with a quick (cluster 2) or a slow (cluster 4) decrease of the first wave. In contrast, patients with a high first wave were clustered in a manner that separated those with a quick (cluster 1), medium (cluster 3) or slow (cluster 5) decrease of the first wave. In the model from Dietz et al*.* [22], the first peak of parasitaemia captured the strength of the innate immune response, and the decrease of the first wave captured the strength of the adaptive immune response. In consequence, these results also confirm that the strength of the immune responses among patients varies greatly. The results further highlighted that patients have even more immune response variability during the first wave than the complete parasitaemia profile. This variability is particularly relevant for patients with an initial high parasitaemia density.

For each cluster of the first wave of parasitaemia, the typical dynamics of the first wave of patients were predicted using the model from Dietz et al., and similar predictions were obtained [22]. Moreover, parameterizing the model using five clusters instead of one cluster reduced the error by 53.4%. In comparison, parameterizing the model using 100 clusters (each patient) instead of one cluster reduced the error by 65.8%. This suggests that the clusters are extremely efficient at capturing the variability in the first parasitaemia waves. Consequently, it is most efficient to consider five clusters to capture within-host variation in a simple mechanistic within-host model, and to inform different recovery rates in a population-based model that investigates the impact of interventions that effect the first parasitaemia wave, such as blood-stage vaccine and drugs.

Investigating the distribution of non-parasitaemia attributes across clusters highlighted which non-parasitaemia attributes can potentially impact within-host dynamics. Interestingly, Female patients did not experience malaria infections with low parasitaemia density and short infection length. Variation in the infection length of malaria between genders supports recent studies [38]. The method of inducing infection and the strain used to infect patients did not vary between clusters. Consequently, the results suggest that these factors do not strongly influence the within-host dynamics in naive individuals. Other external attributes, such as age, could not be compared with the clusters due to the lack of data. Nevertheless, age plays an important role in determining parasitaemia dynamics in the field, as when individuals get older, they gradually develop immunity to malaria due to repeated exposure to the parasites. However, in this study, all patients were naïve to malaria. Thus, age would probably be less critical than observed in the field.

The clustering analyses were performed on subsets of patients from the malariatherapy dataset, thus the conclusions are limited to this dataset, noting there is no other fully detailed set for a complementary analysis. There are limitations of the dataset that may affect the conclusions. First, the detection limit of the microscope was high (10 PRBC/µl). As a consequence, infection lengths may be underestimated in this study [9]. Second, in the malariatherapy dataset, all patients were Afro-American adults suffering from neurosyphilis and naïve to malaria [9]. These patients do not represent all populations at risk for *P. falciparum* (for example, children under 5 years old) [1, 5]. Lastly, in the malariatherapy study, patients received suppressive treatment when they exhibited clinical symptoms due to high parasitaemia density [9]. The clustering analysis of the first parasitaemia wave included 100 patients who did not receive suppressive treatment during the first parasitaemia wave. Therefore, this dataset did not include patients experiencing very high parasitaemia density. This means that although severe malaria is infrequent in the general population [1, 5], these patients are not represented in this dataset.

## Conclusions

Clusters of patients that have similar parasitaemia dynamics were identified using key features that captured the strength of the innate and adaptative immune response. Non-parasitaemia attributes, other than the biological sex of the patient, were not associated with variations in the within-host dynamic. Thus, these results confirm that individuals have personalized and variable strengths of both immune responses, which causes inter-patient variation in the within-host dynamics in naïve individuals. The results suggest that by capturing the variability of patients’ immune response, the variability of within-host dynamics could be efficiently captured. That is, the parasitaemia profiles of patients were reasonably predicted by parameterizing previous within-host models to the identified clusters. The identified clusters can be used to include within-host variation in simplified mechanistic within-host models, and to inform categories of patients in population-based models.

## Supplementary Information

**Additional file 1: Figure S1.** Predicted dynamic of the first parasitaemia wave using parameters as in Dietz et al. (2006). Observed and predicted parasitaemia dynamics and the predicted immune responses of patients (**A**) 46, (**B**) 224, and (**C**) 26 when the constants were calculated as defined by Dietz et al. (2006). **Figure S2.** Predicted dynamic of the first wave of parasitaemia using parameters as defined here. Observed and predicted parasitaemia dynamics and the predicted immune responses of patients (**A**) 46, (**B**) 224, and (**C**) 26, when P_m_ was calculated as defined here. **Figure S3.** Variation of non-parasitaemia attributes across clusters of the full parasitaemia profiles. Visualization of the distribution of (**A**) the sex of the patients, (**B**) repressive treatment usage, (**C**) the way patients were infected, and (**D**) the strain used to infect patients among the two clusters of the full parasitaemia profile. **Figure S4.** Variation of patient gametocyte profiles across clusters of the full parasitaemia profiles. Boxplot of the distribution of (**A**) the number of days with detected gametocytes and (**B**) the maximum density of gametocytes of the patients belonging to the two clusters of the full parasitaemia profile. **Figure S5.** Variation of the patient fever profiles across clusters of the full parasitaemia profiles. Boxplot of the distribution of (A) the maximum value of fever and (B) the number of days with fever of the patients belonging to the two clusters of the full parasitaemia profile. **Figure S6.** Variation of non-parasitaemia attributes across clusters of the first wave of parasitaemia. Visualization of the distribution of (**A**) the sex of the patients, (**B**) the way patients were infected, and (**C**) the strain used to infect patients among the fives clusters of the first wave of parasitaemia.

## Data Availability

The data analysed during the current study are publicly available [9]. The cleaned datasets used for the analysis can be requested to the corresponding author.

## Abbreviations

BIC: Bayesian information criterion
IBM: Individual based model
PfEMP1: *Plasmodium falciparum* erythrocyte membrane protein 1
PRBC: Parasitized red blood cells
RBC: Red blood cells
WHO: World health Organization

## References

[1] WHO. World malaria report 2020: 20 years of global progress and challenges. Geneva: World Health Organization. 2020 [cited 2022 Oct 4]. Available from: https://www.who.int/publications/i/item/9789240015791.

[2] Tuteja R (2007). Malaria—an overview. FEBS J.

[3] Cowman AF, Healer J, Marapana D, Marsh K (2016). Malaria: biology and disease. Cell.

[4] Milner DA (2018). Malaria pathogenesis. Cold Spring Harb Perspect Med.

[5] Mackintosh CL, Beeson JG, Marsh K (2004). Clinical features and pathogenesis of severe malaria. Trends Parasitol.

[6] Miller LH, Baruch DI, Marsh K, Doumbo OK (2002). The pathogenic basis of malaria. Nature.

[7] Meibalan E, Marti M (2017). Biology of malaria transmission. Cold Spring Harb Perspect Med.

[8] Ross A, Killeen G, Smith T (2006). Relationships between host infectivity to mosquitoes and asexual parasite density in *Plasmodium falciparum*. Am J Trop Med Hyg.

[9] Collins WE, Jeffery GM (1999). A retrospective examination of sporozoite and trophozoite-induced infections with *Plasmodium falciparum*: development of parasitologic and clinical immunity during primary infection. Am J Trop Med Hyg.

[10] Natama HM, Rovira-Vallbona E, Krit M, Guetens P, Sorgho H, Somé MA (2021). Genetic variation in the immune system and malaria susceptibility in infants: a nested case–control study in Nanoro. Burkina Faso Malar J.

[11] Flori L, Delahaye NF, Iraqi FA, Hernandez-Valladares FF, Rihet P (2005). TNF as a malaria candidate gene: polymorphism-screening and family-based association analysis of mild malaria attack and parasitemia in Burkina Faso. Genes Immun.

[12] de Mendonça VRR, Goncalves MS, Barral-Netto M (2012). The host genetic diversity in malaria infection. J Trop Med.

[13] Hamilton R, Boots M, Paterson S (2005). The effect of host heterogeneity and parasite intragenomic interactions on parasite population structure. Proc Biol Sci.

[14] Sondo P, Derra K, Lefevre T, Diallo-Nakanabo S, Tarngda Z, Zampa O (2019). Genetically diverse *Plasmodium falciparum* infections, within-host competition and symptomatic malaria in humans. Sci Rep.

[15] Ariey F, Hommel D, Le Scanf C, Duchemin JB, Peneau C, Hulin A (2001). Association of severe malaria with a specific *Plasmodium falciparum* genotype in French Guiana. J Infect Dis.

[16] Gupta S, Hill AV, Kwiatkowski D, Greenwood AM, Greenwood BM, Day KP (1994). Parasite virulence and disease patterns in *Plasmodium falciparum* malaria. Proc Natl Acad Sci USA.

[17] Molineaux L, Träuble M, Collins WE, Jeffery GM, Dietz K (2002). Malaria therapy reinoculation data suggest individual variation of an innate immune response and independent acquisition of antiparasitic and antitoxic immunities. Trans R Soc Trop Med Hyg.

[18] Walther M, Woodruff J, Edele F, Jeffries D, Tongren JE, King E (2006). Innate immune responses to human malaria: heterogeneous cytokine responses to blood-stage *Plasmodium falciparum* correlate with parasitological and clinical outcomes. J Immunol.

[19] Modiano D, Chiucchiuini A, Petrarca V, Sirima BS, Luoni G, Roggero MA (1999). Interethnic differences in the humoral response to non-repetitive regions of the *Plasmodium falciparum* circumsporozoite protein. Am J Trop Med Hyg.

[20] Korbel DS, Newman KC, Almeida CR, Davis DM, Riley EM (2005). Heterogeneous human NK cell responses to *Plasmodium falciparum*-infected erythrocytes. J Immunol.

[21] Molineaux L, Diebner HH, Eichner M, Collins WE, Jeffery GM, Dietz K (2001). *Plasmodium falciparum* parasitaemia described by a new mathematical model. Parasitology.

[22] Dietz K, Raddatz G, Molineaux L (2006). Mathematical model of the first wave of *Plasmodium falciparum* asexual parasitemia in non-immune and vaccinated individuals. Am J Trop Med Hyg.

[23] Johnston GL, Smith DL, Fidock DA (2013). Malaria’s missing number: calculating the human component of R0 by a within-host mechanistic model of *Plasmodium falciparum* infection and transmission. PLoS Comput Biol.

[24] Challenger JD, Bruxvoort K, Ghani AC, Okell LC (2017). Assessing the impact of imperfect adherence to artemether-lumefantrine on malaria treatment outcomes using within-host modelling. Nat Commun.

[25] Childs LM, Buckee CO (2015). Dissecting the determinants of malaria chronicity: why within-host models struggle to reproduce infection dynamics. J R Soc Interface.

[26] Paget-McNicol S, Gatton M, Hastings I, Saul A (2002). The *Plasmodium falciparum var* gene switching rate, switching mechanism and patterns of parasite recrudescence described by mathematical modelling. Parasitology.

[27] Gatton ML, Cheng Q (2004). Investigating antigenic variation and other parasite-host interactions in *Plasmodium falciparum* infections in naïve hosts. Parasitology.

[28] Eckhoff PP (2012). *falciparum* infection durations and infectiousness are shaped by antigenic variation and innate and adaptive host immunity in a mathematical model. PLoS ONE.

[29] Camponovo F, Lee T, Russell J (2021). Mechanistic within-host models of the asexual *Plasmodium falciparum* infection: a review and analytical assessment. Malar J.

[30] Ross R (1915). Some a priori pathometric equations. BMJ.

[31] Macdonald G (1957). The epidemiology and control of malaria.

[32] Bershteyn A, Gerardin J, Bridenbecker D, Lorton CW, Bloedow J, Baker RS (2018). Implementation and applications of EMOD, an individual-based multi-disease modeling platform. Pathog Dis.

[33] Gu W, Killeen GF, Mbogo CM, Regens JL, Githure JI, Beier JC (2003). An individual-based model of *Plasmodium falciparum* malaria transmission on the coast of Kenya. Trans R Soc Trop Med Hyg.

[34] Smith T, Killeen GF, Maire N, Ross A, Molineaux L, Tediosi F (2006). Mathematical modeling of the impact of malaria vaccines on the clinical epidemiology and natural history of *Plasmodium falciparum* malaria: overview. Am J Trop Med Hyg.

[35] Fraley C, Raftery AE, Scrucca L, Brendan T, Fop M. Gaussian mixture modelling for model-based clustering, classification, and density estimation. 2018 [cited 2022 Oct 4]. Available from: https://cran.r-project.org/web/packages/mclust/mclust.pdf.

[36] Coelho CH, Doritchamou JYA, Zaidi I, Duffy PE (2017). Advances in malaria vaccine development: report from the 2017 malaria vaccine symposium. NPJ Vaccines.

[37] Kwiatkowski D (1995). Malarial toxins and the regulation of parasite density. Parasitol Today.

[38] Briggs J, Teyssier N, Nankabirwa JI, Rek J, Jagannathan P, Arinaitwe E (2020). Sex-based differences in clearance of chronic *Plasmodium falciparum* infection. eLife.

