# Supplementary material for: Patient variability in the blood-stage dynamics of Plasmodium falciparum captured by clustering historical data
Source: Malar J. 2022 Oct 26;21:300. doi: 10.1186/s12936-022-04317-0 (PMC9608883; doi:10.1186/s12936-022-04317-0)
Supplement: Supplementary file 1 — Additional file 1: Figure S1. Predicted dynamic of the first parasitaemia wave using parameters as in Dietz et al. (2006). Observed and predicted parasitaemia dynamics and the predicted immune responses of patients (A) 46, (B) 224, and (C) 26 when the constants were calculated as defined by Dietz et al. (2006). Figure S2. Predicted dynamic of the first wave of parasitaemia using parameters as defined here. Observed and predicted parasitaemia dynamics and the predicted immune responses of patients (A) 46, (B) 224, and (C) 26, when Pm was calculated as defined here. Figure S3. Variation of non-parasitaemia attributes across clusters of the full parasitaemia profiles. Visualization of the distribution of (A) the sex of the patients, (B) repressive treatment usage, (C) the way patients were infected, and (D) the strain used to infect patients among the two clusters of the full parasitaemia profile. Figure S4. Variation of patient gametocyte profiles across clusters of the full parasitaemia profiles. Boxplot of the distribution of (A) the number of days with detected gametocytes and (B) the maximum density of gametocytes of the patients belonging to the two clusters of the full parasitaemia profile. Figure S5. Variation of the patient fever profiles across clusters of the full parasitaemia profiles. Boxplot of the distribution of (A) the maximum value of fever and (B) the number of days with fever of the patients belonging to the two clusters of the full parasitaemia profile. Figure S6. Variation of non-parasitaemia attributes across clusters of the first wave of parasitaemia. Visualization of the distribution of (A) the sex of the patients, (B) the way patients were infected, and (C) the strain used to infect patients among the fives clusters of the first wave of parasitaemia. [file 12936_2022_4317_MOESM1_ESM.docx]

Patient variability in the blood-stage dynamics of *Plasmodium falciparum* captured by clustering historical data

**Additional file 1**

Thiery Masserey^1,2,*^, Melissa A Penny^1,2^,Tamsin Lee^1,2^

^1^Swiss Tropical and Public Health Institute, Allschwil, Switzerland

^2^University of Basel, Basel, Switzerland

^*^Correspondence: [thiery.masserey@swisstph.ch](mailto:thiery.masserey@swisstph.ch)

# Simplification of the model of Dietz et al. (2006)

The model from Dietz *et al.* (2006) used four case-specific constants. However, Dietz *et al.* (2006) used a Powell hill climbing algorithm to calculate the values of these constants instead of calculating them as defined (Table 4). First, the model of Dietz *et al.* (2006) was replicated, and the case-specific constants were calculated as defined by Dietz *et al.* (2006). With this parameterization, the model captured the increase of the different first parasitaemia waves but not its decrease (Figure S1). It was because the adaptive immune responses were either too strong (Figure S1C) or too weak (Figures S1A and B).

Figure S1: Predicted dynamic of the first parasitaemia wave using parameters as in Dietz et al. (2006)

Observed and predicted parasitaemia dynamics and the predicted immune responses of patients (**A**) 46, (**B**) 224, and (**C**) 26 when the constants were calculated as defined by Dietz et al. (2006).


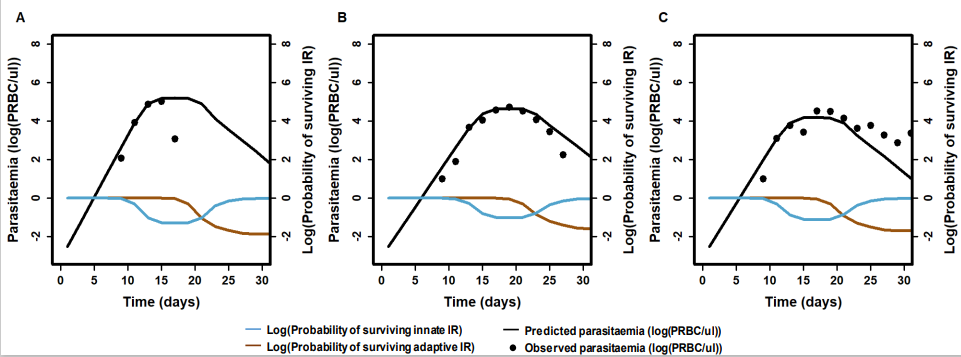


For this reason, the definition of the cumulative effective density, *P_m_** (that captured the strength of the adaptive immune response) was modified. Its was defined as a constant divided by the ratio between the maximum density and the last density of the first parasitaemia wave. Setting the constant to 4830 gave the closest prediction of the patient data (Figure S2), as found by trial and error. In addition, the dynamics of the immune responses of each patient agreed with Dietz *et al.* (2006).

Figure S2: Predicted dynamic of the first wave of parasitaemia using parameters as defined here

Observed and predicted parasitaemia dynamics and the predicted immune responses of patients (**A**) 46, (**B**) 224, and (**C**) 26, when P_m_* was calculated as defined here.


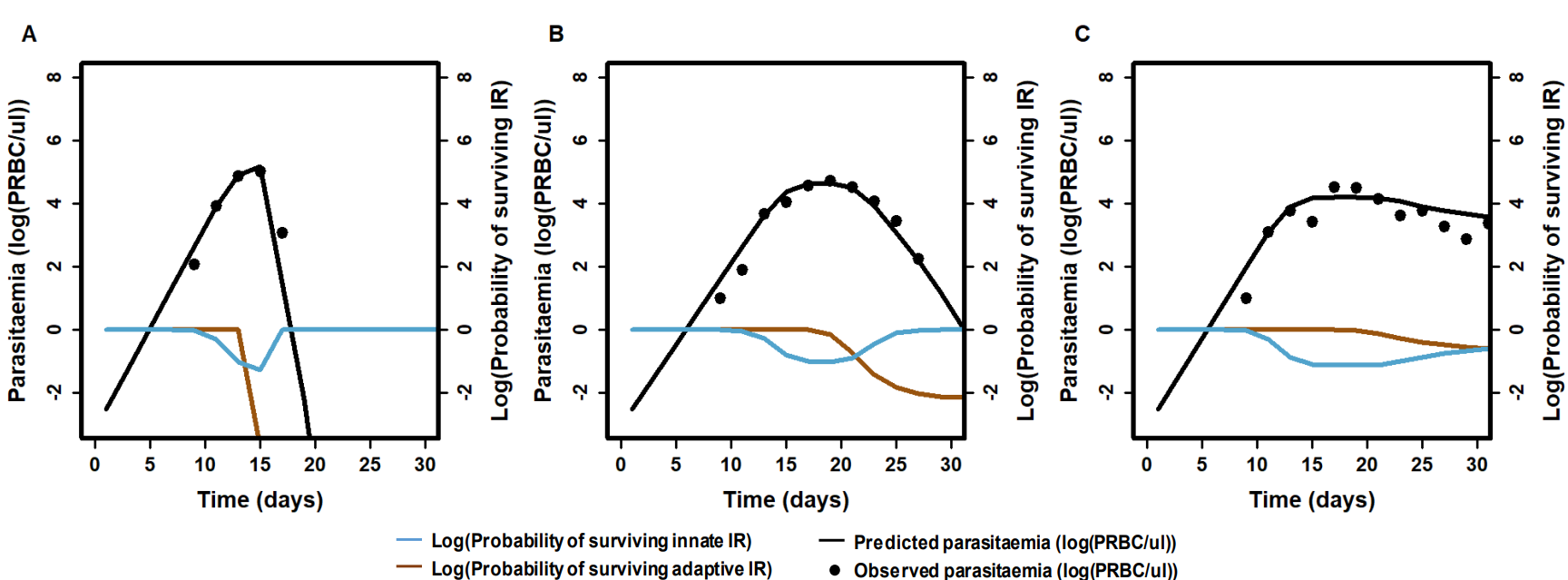


#### Variation of non-parasitaemia attributes across clusters

Figure S3: Variation of non-parasitaemia attributes across clusters of the full parasitaemia profiles

Visualization of the distribution of (**A**) the sex of the patients, (**B**) repressive treatment usage, (**C**) the way patients were infected, and (**D**) the strain used to infect patients among the two clusters of the full parasitaemia profile


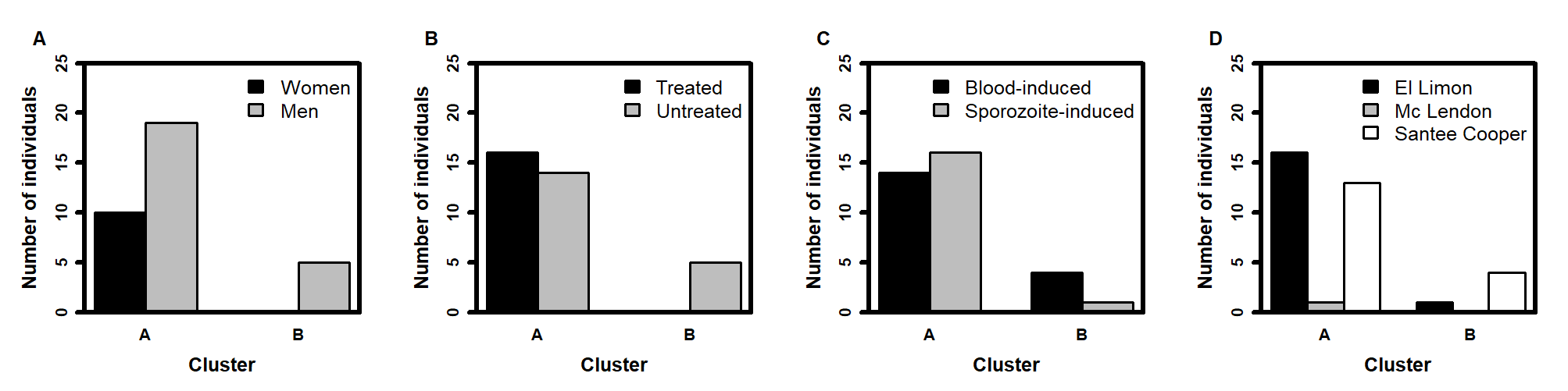


.

Figure S4: Variation of patient gametocyte profiles across clusters of the full parasitaemia profiles

Boxplot of the distribution of (**A**) the number of days with detected gametocytes and (**B**) the maximum density of gametocytes of the patients belonging to the two clusters of the full parasitaemia profile.


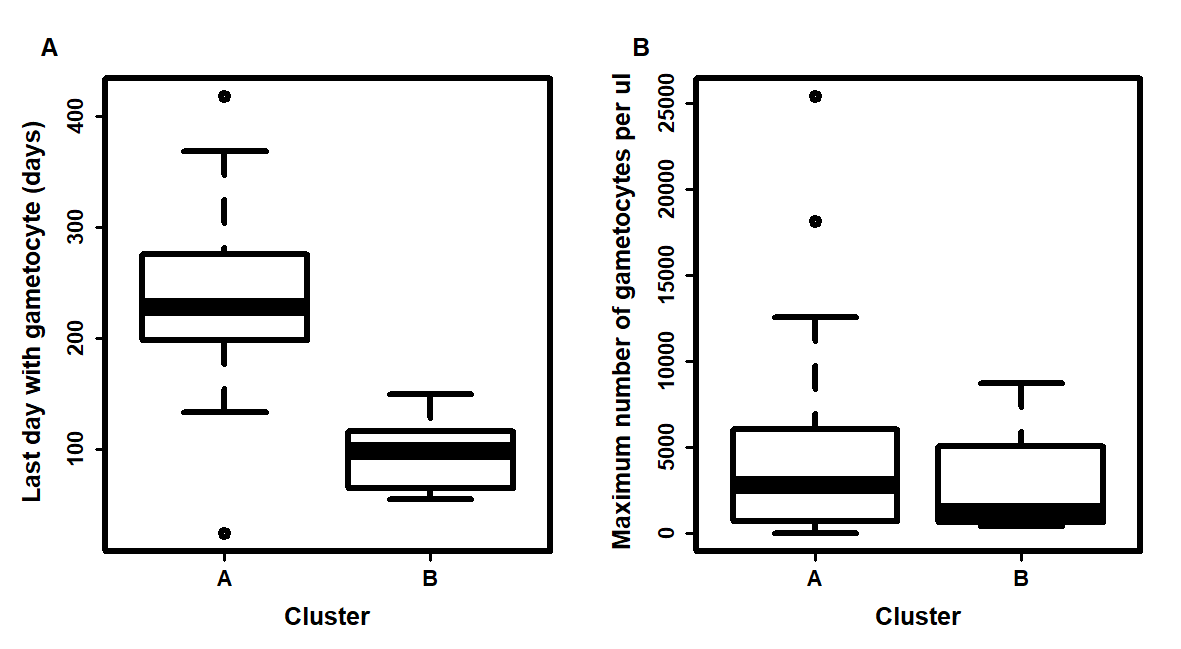


Figure S5: Variation of the patient fever profiles across clusters of the full parasitaemia profiles


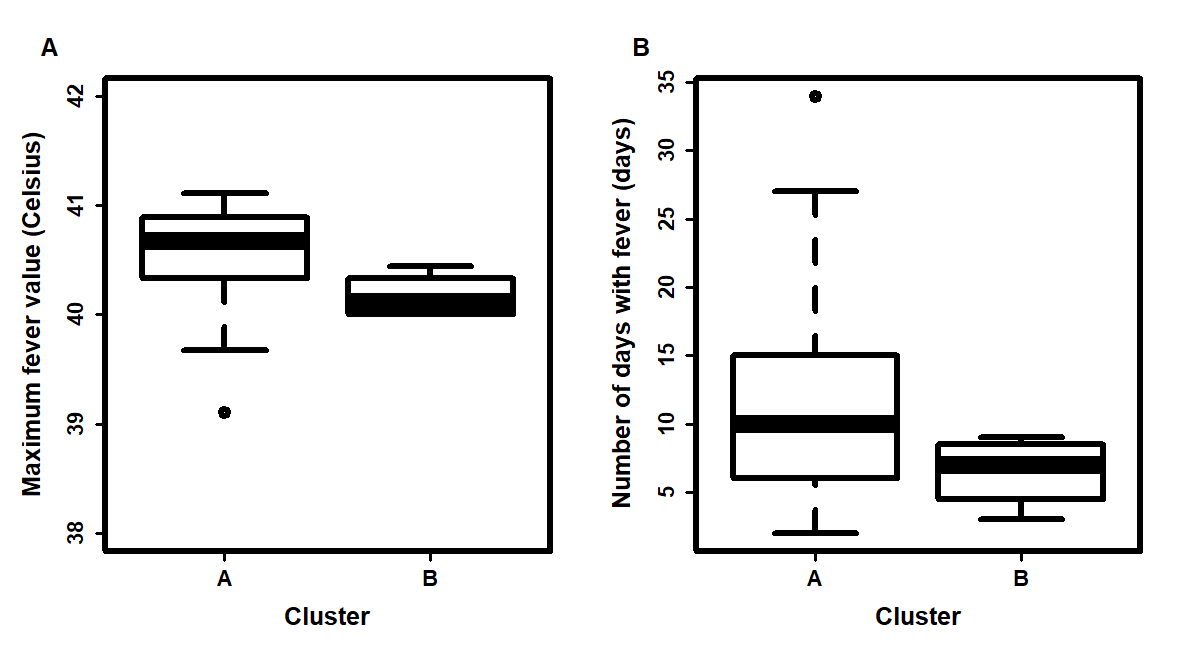
Boxplot of the distribution of (A) the maximum value of fever and (B) the number of days with fever of the patients belonging to the two clusters of the full parasitaemia profile.

Figure S6: Variation of non-parasitaemia attributes across clusters of the first wave of parasitaemia.


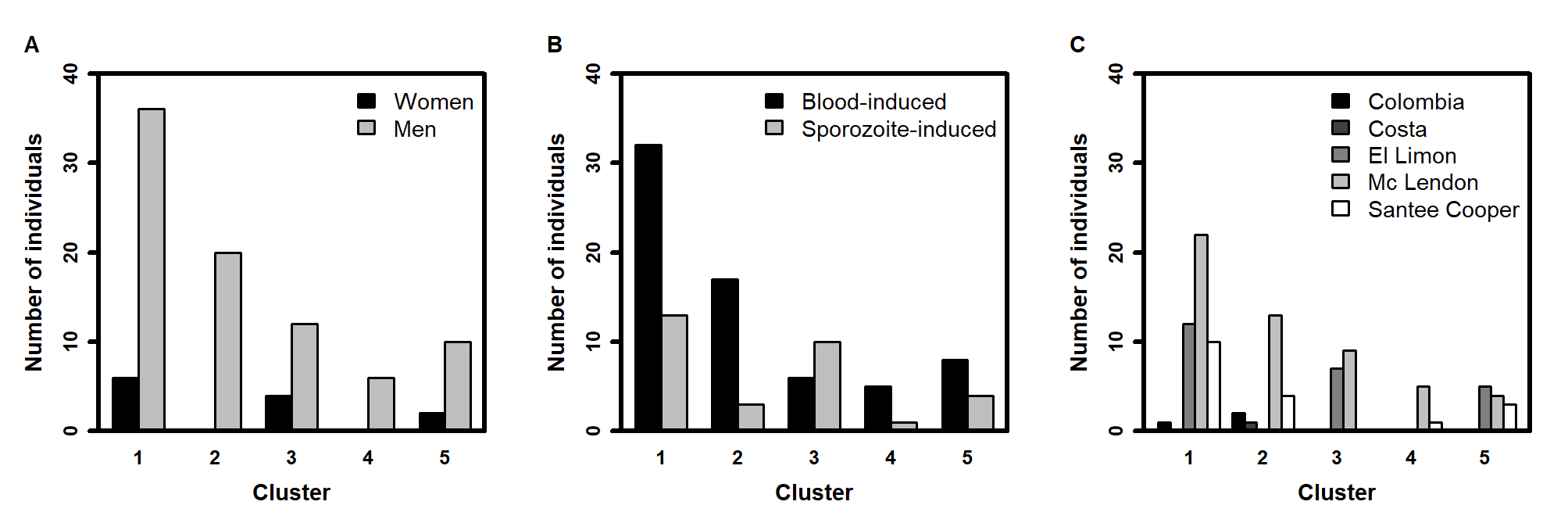
Visualization of the distribution of (**A**) the sex of the patients, (**B**) the way patients were infected, and (**C**) the strain used to infect patients among the fives clusters of the first wave of parasitaemia.
